# Bis[μ-4-methyl-2-(2-pyridyl­methyl­sulfan­yl)pyrimidine-κ*N* ^1^]bis­[(trifluoro­methanesulfonato-κ*O*)silver(I)]

**DOI:** 10.1107/S1600536810043631

**Published:** 2010-10-31

**Authors:** Huan-Huan Wang, Chao-Yan Zhang, Yue Cui, Qian Gao, Ya-Bo Xie

**Affiliations:** a College of Environmental and Energy Engineering, Beijing University of Technology, Beijing 100124, People’s Republic of China

## Abstract

In the centrosymmetric dinuclear title complex, [Ag_2_(CF_3_SO_3_)_2_(C_11_H_11_N_3_S)_2_], the Ag^I^ atom is coordinated by two N atoms from two 4-methyl-2-(2-pyridyl­methyl­sulfan­yl)pyrimidine ligands and one O atom from a trifluoro­methane­sulfonate anion in a distorted T-type coordination geometry. The ligand adopts a bidentate bridging coordination mode through one pyridyl N atom and one pyrimidine N atom. In the crystal structure, π–π inter­actions are present between adjacent pyrimidine rings, with a centroid-to-centroid distance of 3.875 (7) Å.

## Related literature

For the architectures of metal complexes, see: Hamblin *et al.* (2002 ▶). For a related structure, see: Xie *et al.* (2006 ▶).
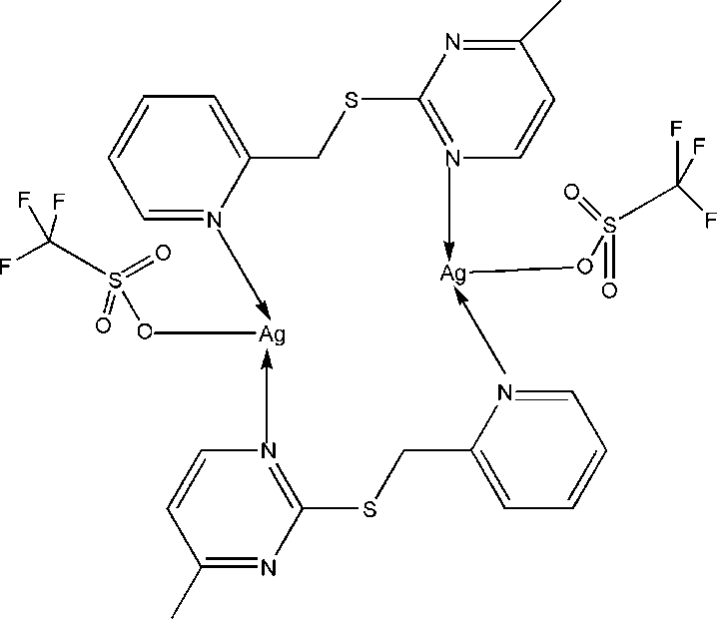


## Experimental

### 

#### Crystal data

[Ag_2_(CF_3_O_3_S)_2_(C_11_H_11_N_3_S)_2_]
*M* *_r_* = 948.50
Triclinic, 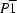

*a* = 8.9999 (18) Å
*b* = 9.1087 (18) Å
*c* = 10.937 (2) Å
α = 75.07 (3)°
β = 88.59 (3)°
γ = 68.97 (3)°
*V* = 806.3 (3) Å^3^
*Z* = 1
Mo *K*α radiation
μ = 1.56 mm^−1^
*T* = 293 K
0.15 × 0.12 × 0.10 mm

#### Data collection

Bruker APEX CCD diffractometer
Absorption correction: multi-scan (*SADABS*; Bruker, 2001 ▶) *T*_min_ = 0.800, *T*_max_ = 0.860
8621 measured reflections
3681 independent reflections
3007 reflections with *I* > 2σ(*I*)
*R*_int_ = 0.028

#### Refinement

*R*[*F* ^2^ > 2σ(*F* ^2^)] = 0.044
*wR*(*F* ^2^) = 0.138
*S* = 0.97
3681 reflections
217 parameters
H-atom parameters constrained
Δρ_max_ = 0.72 e Å^−3^
Δρ_min_ = −0.41 e Å^−3^

### 

Data collection: *SMART* (Bruker, 2007 ▶); cell refinement: *SAINT* (Bruker, 2007 ▶); data reduction: *SAINT*; program(s) used to solve structure: *SHELXS97* (Sheldrick, 2008 ▶); program(s) used to refine structure: *SHELXL97* (Sheldrick, 2008 ▶); molecular graphics: *SHELXTL* (Sheldrick, 2008 ▶); software used to prepare material for publication: *SHELXTL*.

## Supplementary Material

Crystal structure: contains datablocks I, global. DOI: 10.1107/S1600536810043631/hy2366sup1.cif

Structure factors: contains datablocks I. DOI: 10.1107/S1600536810043631/hy2366Isup2.hkl

Additional supplementary materials:  crystallographic information; 3D view; checkCIF report

## Figures and Tables

**Table 1:** Selected bond lengths (Å)

Ag1—N1	2.150 (4)
Ag1—N3^i^	2.161 (3)
Ag1—O5	2.700 (4)

Symmetry code: (i) 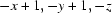.

**Fig. 1.**
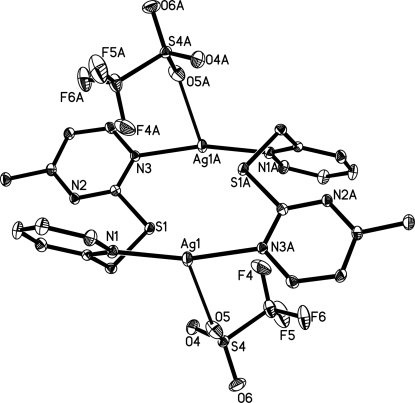
The molecular structure of the title compound, with displacement ellipsoids drawn at the 30% probability level. [Symmetry code: (A) -x + 1, -y + 1, -z.]

## supplementary crystallographic information

### Comment

The coordination geometry of metal ions and the nature of ligands decide the generation of coordination architectures (Hamblin *et al.*, 2002). In previous studies, much attention has been paid to the use of flexible bridging ligands because of their conformational freedom and flexible properties (Xie *et al.*, 2006). As part of our investigation of flexible ligands and their complexes, the crystal structure of a silver(I) complex with a flexible thioether ligand, the title compound, is reported here.

In the binuclear structure of the title complex (Fig. 1), the Ag^I^ atom is coordinated by two N atoms from two 4-methyl-2-(2-pyridylmethylsulfanyl)pyrimidine ligands and one O atom from a trifluoromethanesulfonate anion (Table 1), displaying a slightly distorted T-type coordination geometry. The ligand adopts a bidentate bridging coordination mode through two N atoms. The dihedral angle between the pyrimidine ring and pyridine ring is 82.67 (3)°. The two pyrimidine rings are nearly parallel, and so are the two pyridine rings. In the crystal structure, π–π interactions between adjacent pyrimidine rings are present, with a centroid–centroid distance of 3.875 (7) Å.

### Experimental

A solution of AgSO_3_CF_3_ (0.04 mmol) in acetone (4 ml) was carefully layered on top of a mixture of chloroform (2 ml) and acetone (2 ml), which was carefully layered on top of a solution of 4-methyl-2-(2-pyridylmethylsulfanyl)pyrimidine (0.04 mmol) in chloroform (4 ml) in a test tube. After 2 weeks at room temperature, colourless prism single crystals appeared.

### Refinement

All H atoms were positioned geometrically and refined as riding, with C—H = 0.93–0.97 Å and *U*_iso_(H) = 1.2(1.5 for methyl)*U*_eq_(C).

### Crystal data

**Table:**

[Ag_2_(CF_3_O_3_S)_2_(C_11_H_11_N_3_S)_2_]	*Z* = 1
*M**_r_* = 948.50	*F*(000) = 468
Triclinic, *P*1	*D*_x_ = 1.953 Mg m^−^^3^
Hall symbol: -P 1	Mo *K*α radiation, λ = 0.71073 Å
*a* = 8.9999 (18) Å	Cell parameters from 3696 reflections
*b* = 9.1087 (18) Å	θ = 3.1–27.5°
*c* = 10.937 (2) Å	µ = 1.56 mm^−^^1^
α = 75.07 (3)°	*T* = 293 K
β = 88.59 (3)°	Prism, colourless
γ = 68.97 (3)°	0.15 × 0.12 × 0.10 mm
*V* = 806.3 (3) Å^3^	

### Data collection

**Table:**

Bruker APEX CCD diffractometer	3681 independent reflections
Radiation source: fine-focus sealed tube	3007 reflections with *I* > 2σ(*I*)
graphite	*R*_int_ = 0.028
φ and ω scans	θ_max_ = 27.5°, θ_min_ = 3.1°
Absorption correction: multi-scan (*SADABS*; Bruker, 2001)	*h* = −11→11
*T*_min_ = 0.800, *T*_max_ = 0.860	*k* = −11→11
8621 measured reflections	*l* = −14→14

### Refinement

**Table:**

Refinement on *F*^2^	Primary atom site location: structure-invariant direct methods
Least-squares matrix: full	Secondary atom site location: difference Fourier map
*R*[*F*^2^ > 2σ(*F*^2^)] = 0.044	Hydrogen site location: inferred from neighbouring sites
*wR*(*F*^2^) = 0.138	H-atom parameters constrained
*S* = 0.97	*w* = 1/[σ^2^(*F*_o_^2^) + (0.1*P*)^2^] where *P* = (*F*_o_^2^ + 2*F*_c_^2^)/3
3681 reflections	(Δ/σ)_max_ = 0.001
217 parameters	Δρ_max_ = 0.72 e Å^−^^3^
0 restraints	Δρ_min_ = −0.41 e Å^−^^3^

### Fractional atomic coordinates and isotropic or equivalent isotropic displacement parameters (Å^2^)

**Table:**

	*x*	*y*	*z*	*U*_iso_*/*U*_eq_	
Ag1	0.76401 (4)	0.50847 (4)	0.08737 (3)	0.04428 (16)	
S1	0.60205 (13)	0.26916 (13)	0.01310 (10)	0.0376 (3)	
N1	0.7984 (4)	0.2971 (4)	0.2435 (3)	0.0379 (8)	
N2	0.5373 (4)	0.0216 (4)	0.1602 (3)	0.0348 (7)	
N3	0.3225 (4)	0.2503 (4)	0.0425 (3)	0.0318 (7)	
C1	0.8050 (6)	0.3150 (6)	0.3602 (4)	0.0463 (11)	
H1A	0.8072	0.4131	0.3690	0.056*	
C2	0.8086 (6)	0.1969 (6)	0.4671 (5)	0.0526 (12)	
H2A	0.8122	0.2143	0.5470	0.063*	
C3	0.8067 (7)	0.0511 (6)	0.4541 (5)	0.0540 (12)	
H3A	0.8082	−0.0316	0.5253	0.065*	
C4	0.8026 (5)	0.0294 (5)	0.3361 (5)	0.0440 (10)	
H4A	0.8026	−0.0689	0.3261	0.053*	
C5	0.7986 (5)	0.1535 (5)	0.2314 (4)	0.0340 (8)	
C6	0.7891 (5)	0.1365 (5)	0.1003 (4)	0.0357 (9)	
H6A	0.8038	0.0243	0.1048	0.043*	
H6B	0.8751	0.1606	0.0551	0.043*	
C7	0.4768 (5)	0.1670 (5)	0.0809 (4)	0.0314 (8)	
C8	0.4366 (5)	−0.0528 (5)	0.2077 (4)	0.0374 (9)	
C9	0.2748 (5)	0.0237 (5)	0.1742 (5)	0.0420 (10)	
H9A	0.2036	−0.0269	0.2068	0.050*	
C10	0.2224 (5)	0.1756 (5)	0.0921 (5)	0.0420 (10)	
H10A	0.1136	0.2291	0.0697	0.050*	
C11	0.5077 (6)	−0.2182 (6)	0.2968 (5)	0.0533 (12)	
H11A	0.6214	−0.2489	0.3064	0.080*	
H11B	0.4831	−0.2954	0.2638	0.080*	
H11C	0.4645	−0.2171	0.3779	0.080*	
S4	0.93636 (13)	0.38405 (13)	−0.22242 (10)	0.0377 (3)	
F4	0.6405 (4)	0.5740 (6)	−0.2672 (5)	0.1020 (14)	
F5	0.7551 (7)	0.5239 (5)	−0.4298 (4)	0.130 (2)	
F6	0.8009 (6)	0.6879 (4)	−0.3447 (5)	0.1096 (16)	
O4	0.8891 (5)	0.2489 (4)	−0.2085 (4)	0.0680 (11)	
O5	0.9355 (5)	0.4366 (5)	−0.1096 (3)	0.0722 (12)	
O6	1.0735 (5)	0.3760 (5)	−0.2887 (5)	0.0839 (15)	
C24	0.7756 (8)	0.5505 (7)	−0.3235 (5)	0.0635 (15)	

### Atomic displacement parameters (Å^2^)

**Table:**

	*U*^11^	*U*^22^	*U*^33^	*U*^12^	*U*^13^	*U*^23^
Ag1	0.0506 (2)	0.0315 (2)	0.0441 (2)	−0.01457 (15)	−0.00205 (16)	0.00090 (15)
S1	0.0406 (6)	0.0352 (5)	0.0372 (6)	−0.0193 (4)	0.0008 (4)	−0.0018 (4)
N1	0.0400 (19)	0.0379 (19)	0.0376 (19)	−0.0195 (16)	0.0016 (15)	−0.0051 (15)
N2	0.0354 (17)	0.0285 (16)	0.0391 (19)	−0.0131 (14)	−0.0017 (15)	−0.0044 (14)
N3	0.0337 (17)	0.0267 (16)	0.0347 (17)	−0.0119 (13)	0.0004 (14)	−0.0065 (13)
C1	0.062 (3)	0.041 (2)	0.044 (3)	−0.028 (2)	0.003 (2)	−0.011 (2)
C2	0.067 (3)	0.052 (3)	0.044 (3)	−0.029 (2)	−0.001 (2)	−0.011 (2)
C3	0.076 (3)	0.044 (3)	0.040 (3)	−0.030 (3)	−0.001 (2)	0.005 (2)
C4	0.045 (2)	0.033 (2)	0.055 (3)	−0.0189 (19)	−0.004 (2)	−0.006 (2)
C5	0.0302 (19)	0.0310 (19)	0.039 (2)	−0.0112 (16)	0.0035 (17)	−0.0058 (17)
C6	0.030 (2)	0.036 (2)	0.043 (2)	−0.0138 (16)	0.0022 (17)	−0.0113 (18)
C7	0.035 (2)	0.0287 (19)	0.033 (2)	−0.0123 (16)	0.0003 (16)	−0.0118 (16)
C8	0.048 (2)	0.0286 (19)	0.038 (2)	−0.0159 (18)	0.0007 (19)	−0.0090 (17)
C9	0.041 (2)	0.037 (2)	0.052 (3)	−0.0249 (19)	0.001 (2)	−0.004 (2)
C10	0.030 (2)	0.038 (2)	0.055 (3)	−0.0130 (17)	−0.0072 (19)	−0.007 (2)
C11	0.053 (3)	0.040 (2)	0.059 (3)	−0.019 (2)	−0.002 (2)	0.003 (2)
S4	0.0418 (6)	0.0342 (5)	0.0405 (6)	−0.0173 (4)	0.0089 (5)	−0.0110 (4)
F4	0.053 (2)	0.114 (3)	0.111 (3)	0.003 (2)	−0.013 (2)	−0.029 (3)
F5	0.206 (6)	0.096 (3)	0.053 (2)	−0.003 (4)	−0.048 (3)	−0.029 (2)
F6	0.160 (4)	0.0394 (19)	0.110 (3)	−0.030 (2)	−0.016 (3)	0.005 (2)
O4	0.073 (3)	0.0426 (19)	0.096 (3)	−0.0370 (19)	0.015 (2)	−0.009 (2)
O5	0.079 (3)	0.071 (3)	0.050 (2)	0.002 (2)	−0.015 (2)	−0.029 (2)
O6	0.072 (3)	0.068 (3)	0.121 (4)	−0.036 (2)	0.055 (3)	−0.030 (3)
C24	0.092 (4)	0.046 (3)	0.040 (3)	−0.010 (3)	−0.010 (3)	−0.011 (2)

### Geometric parameters (Å, °)

**Table:**

Ag1—N1	2.150 (4)	C4—H4A	0.9300
Ag1—N3^i^	2.161 (3)	C5—C6	1.488 (6)
Ag1—O5	2.700 (4)	C6—H6A	0.9700
S1—C7	1.744 (4)	C6—H6B	0.9700
S1—C6	1.797 (4)	C8—C9	1.381 (6)
N1—C1	1.333 (6)	C8—C11	1.488 (6)
N1—C5	1.347 (5)	C9—C10	1.362 (6)
N2—C7	1.311 (5)	C9—H9A	0.9300
N2—C8	1.337 (5)	C10—H10A	0.9300
N3—C7	1.337 (5)	C11—H11A	0.9600
N3—C10	1.340 (5)	C11—H11B	0.9600
N3—Ag1^i^	2.161 (3)	C11—H11C	0.9600
C1—C2	1.362 (7)	S4—O6	1.404 (4)
C1—H1A	0.9300	S4—O4	1.412 (3)
C2—C3	1.378 (7)	S4—O5	1.433 (4)
C2—H2A	0.9300	S4—C24	1.804 (6)
C3—C4	1.358 (7)	F4—C24	1.323 (8)
C3—H3A	0.9300	F5—C24	1.278 (6)
C4—C5	1.377 (6)	F6—C24	1.313 (7)
			
N1—Ag1—N3^i^	164.97 (13)	N2—C7—N3	126.4 (4)
N1—Ag1—O5	112.81 (13)	N2—C7—S1	119.9 (3)
N3^i^—Ag1—O5	81.84 (13)	N3—C7—S1	113.7 (3)
C7—S1—C6	100.93 (19)	N2—C8—C9	120.3 (4)
C1—N1—C5	118.1 (4)	N2—C8—C11	116.8 (4)
C1—N1—Ag1	117.2 (3)	C9—C8—C11	122.9 (4)
C5—N1—Ag1	124.4 (3)	C10—C9—C8	118.0 (4)
C7—N2—C8	117.6 (3)	C10—C9—H9A	121.0
C7—N3—C10	115.5 (3)	C8—C9—H9A	121.0
C7—N3—Ag1^i^	123.1 (3)	N3—C10—C9	122.2 (4)
C10—N3—Ag1^i^	121.4 (3)	N3—C10—H10A	118.9
N1—C1—C2	123.3 (4)	C9—C10—H10A	118.9
N1—C1—H1A	118.4	C8—C11—H11A	109.5
C2—C1—H1A	118.4	C8—C11—H11B	109.5
C1—C2—C3	118.3 (5)	H11A—C11—H11B	109.5
C1—C2—H2A	120.8	C8—C11—H11C	109.5
C3—C2—H2A	120.8	H11A—C11—H11C	109.5
C4—C3—C2	119.3 (5)	H11B—C11—H11C	109.5
C4—C3—H3A	120.4	O6—S4—O4	115.1 (3)
C2—C3—H3A	120.4	O6—S4—O5	113.5 (3)
C3—C4—C5	119.8 (4)	O4—S4—O5	115.0 (3)
C3—C4—H4A	120.1	O6—S4—C24	105.0 (3)
C5—C4—H4A	120.1	O4—S4—C24	103.5 (3)
N1—C5—C4	121.2 (4)	O5—S4—C24	102.9 (2)
N1—C5—C6	117.4 (4)	S4—O5—Ag1	144.3 (3)
C4—C5—C6	121.4 (4)	F5—C24—F6	108.9 (5)
C5—C6—S1	112.7 (3)	F5—C24—F4	108.0 (6)
C5—C6—H6A	109.0	F6—C24—F4	106.2 (5)
S1—C6—H6A	109.0	F5—C24—S4	112.2 (4)
C5—C6—H6B	109.0	F6—C24—S4	111.1 (5)
S1—C6—H6B	109.0	F4—C24—S4	110.1 (4)
H6A—C6—H6B	107.8		
			
N3^i^—Ag1—N1—C1	−51.9 (6)	C6—S1—C7—N2	−7.9 (4)
O5—Ag1—N1—C1	141.6 (3)	C6—S1—C7—N3	172.8 (3)
N3^i^—Ag1—N1—C5	121.5 (5)	C7—N2—C8—C9	−0.7 (6)
O5—Ag1—N1—C5	−45.0 (4)	C7—N2—C8—C11	179.6 (4)
C5—N1—C1—C2	−1.4 (7)	N2—C8—C9—C10	0.1 (7)
Ag1—N1—C1—C2	172.5 (4)	C11—C8—C9—C10	179.8 (4)
N1—C1—C2—C3	0.6 (8)	C7—N3—C10—C9	−1.2 (6)
C1—C2—C3—C4	0.5 (8)	Ag1^i^—N3—C10—C9	−179.9 (4)
C2—C3—C4—C5	−0.8 (8)	C8—C9—C10—N3	0.9 (7)
C1—N1—C5—C4	1.1 (6)	O6—S4—O5—Ag1	177.9 (4)
Ag1—N1—C5—C4	−172.3 (3)	O4—S4—O5—Ag1	−46.7 (5)
C1—N1—C5—C6	179.2 (4)	C24—S4—O5—Ag1	65.0 (5)
Ag1—N1—C5—C6	5.8 (5)	N1—Ag1—O5—S4	85.8 (4)
C3—C4—C5—N1	0.0 (7)	N3^i^—Ag1—O5—S4	−90.7 (4)
C3—C4—C5—C6	−178.1 (4)	O6—S4—C24—F5	62.5 (6)
N1—C5—C6—S1	−65.9 (4)	O4—S4—C24—F5	−58.5 (6)
C4—C5—C6—S1	112.3 (4)	O5—S4—C24—F5	−178.5 (5)
C7—S1—C6—C5	−76.0 (3)	O6—S4—C24—F6	−59.7 (5)
C8—N2—C7—N3	0.5 (6)	O4—S4—C24—F6	179.3 (4)
C8—N2—C7—S1	−178.8 (3)	O5—S4—C24—F6	59.2 (5)
C10—N3—C7—N2	0.5 (6)	O6—S4—C24—F4	−177.1 (4)
Ag1^i^—N3—C7—N2	179.2 (3)	O4—S4—C24—F4	61.8 (4)
C10—N3—C7—S1	179.7 (3)	O5—S4—C24—F4	−58.2 (5)
Ag1^i^—N3—C7—S1	−1.6 (4)		

Symmetry codes: (i) −*x*+1, −*y*+1, −*z*.
